# Maternal Flavonoids Intake Reverts Depression-Like Behaviour in Rat Female Offspring

**DOI:** 10.3390/nu11030572

**Published:** 2019-03-07

**Authors:** Ana Laura de la Garza, Miguel A. Garza-Cuellar, Ivan A. Silva-Hernandez, Robbi E. Cardenas-Perez, Luis A. Reyes-Castro, Elena Zambrano, Brenda Gonzalez-Hernandez, Lourdes Garza-Ocañas, Lizeth Fuentes-Mera, Alberto Camacho

**Affiliations:** 1 Centro de Investigación en Nutrición y Salud Pública, Facultad de Salud Pública y Nutrición, Universidad Autonoma de Nuevo Leon, 64460 Monterrey, Mexico; ana.dlgarzah@uanl.mx; 2 Nutrition Unit, Center for Research and Development in Health Sciences, Universidad Autonoma de Nuevo Leon, 64460 Monterrey, Mexico; 3 Department of Biochemistry, Facultad de Ciencias Biologicas, Universidad Autonoma de Nuevo Leon, 66455 Monterrey, Mexico; miguel_94.10@hotmail.com (M.A.G.-C.); ivan_blaze914@hotmail.com (I.A.S.-H.); gonzalebrenda@yahoo.com (B.G.-H.); 4 Department of Biochemistry, Facultad de Medicina, Universidad Autonoma de Nuevo Leon, 64460 Monterrey, Mexico; robbielizac@gmail.com (R.E.C.-P.); lizeth46@hotmail.com (L.F.-M.); 5 Neurometabolism Unit, Center for Research and Development in Health Sciences, Universidad Autonoma de Nuevo Leon, 64460 Monterrey, Mexico; 6 Reproductive Biology Department, Instituto Nacional de Ciencias Medicas y Nutrición Salvador Zubiran, 14080 Mexico City, Mexico; lafe_mat@hotmail.com (L.A.R.-C.); zamgon@yahoo.com.mx (E.Z.); 7 Department of Pharmacology, Facultad de Medicina, Universidad Autonoma de Nuevo Leon, 64460 Monterrey, Mexico; ogarza@live.com.mx

**Keywords:** depression, nutritional programming, kaempferol, narirutin, inflammation

## Abstract

Maternal hypercaloric exposure during pregnancy and lactation is a risk factor for developing diseases associated with inflammation such as obesity, diabetes and, neurological diseases in the offspring. Neuroinflammation might modulate neuronal activation and flavonoids are dietary compounds that have been proven to exert anti-inflammatory properties. Thus, the aim of the present study is to evaluate the effect of maternal supplementation with flavonoids (kaempferol-3-*O*-glucoside and narirutin) on the prevention of depression-like behaviour in the female offspring of dams fed with an obesogenic diet during the perinatal period. Maternal programming was induced by high fat (HFD), high sugar (HSD), or cafeteria diets exposure and depressive like-behaviour, referred to as swimming, climbing, and immobility events, was evaluated around postnatal day 56–60 before and after 30 mg/kg i.p. imipramine administration in the female offspring groups. Central inflammation was analyzed by measuring the TANK binding kinase 1 (TBK1) expression. We found that the offspring of mothers exposed to HSD programming failed to show the expected antidepressant effect of imipramine. Also, imipramine injection, to the offspring of mothers exposed to cafeteria diet, displayed a pro-depressive like-behaviour phenotype. However, dietary supplementation with flavonoids reverted the depression-like behaviour in the female offspring. Finally, we found that HSD programming increases the TBK1 inflammatory protein marker in the hippocampus. Our data suggest that maternal HSD programming disrupts the antidepressant effect of imipramine whereas cafeteria diet exposure leads to depressive-like behaviour in female offspring, which is reverted by maternal flavonoid supplementation.

## 1. Introduction

Depression affects more than 300 million people worldwide and is one of the leading causes of disability (World Health Organization). Maternal overnutrition disrupts the peripheral and central metabolic homeostasis and increases the susceptibility to behaviour alterations in humans and animal models [1,2]. For instance, hypercaloric diet exposure, such as high fat diet (HFD), cafeteria diet (CAF), and high sugar diet (HSD), intake in rodents programs offspring to show long-term behavioral defects later in life, including anxiety, autism, addiction-like, and depression-like behavior [3,4,5] Potential molecular changes on these scenarios are associated with the disruption of dopamine and opioid neurotransmission in the nucleus accumbens and the prefrontal cortex [6,7], increased circulating corticosterone plasma levels [8,9], oxidative stress [9] and peripheral and central inflammation [4].

The role of the immune system and brain function during metabolic programming have started to decode. Maternal programming by hypercaloric diet exposure sets a peripheral and central inflammation which modulates defective behaviour in the offspring, favoring depression-like behaviour phenotypes in murine models [10,11,12,13,14]. Substantial evidence has documented a positive proinflammatory profile in patients with idiopathic major depression including increases in circulating proinflammatory cytokines, interleukin (IL)-1, IL-6, tumor necrosis factor (TNF)-alpha, and C-reactive protein (CRP) [15], which correlates with depression severity [15,16]. Molecularly, IL-6 and TNF-α production is in part modulated by the NF-κB pathway, which is associated to the IκB kinase (IKK)-related kinase family, where the TANK binding kinase 1 (TBK1) is an important member of the node [17,18]. The NF-κB-TBK1 pathway is activated by lipopolysaccharides and saturated fatty acids by TLR4-dependent signaling cascade [17,18]. Of note, central TLR4 activation by saturated fatty acids leads to inflammatory response and metabolic complications [17,18]. We have previously reported that TBK1 is also activated by saturated fatty acids and it is translocated to the plasma membrane in a rodent model of obesity [19]. Hypercaloric programming during pregnancy sets a peripheral and central inflammatory profile leading to metabolic complications and depression susceptibility in offspring.

Flavonoids, non-nutritive components found mainly in the plant kingdom, regulate cell physiology by negatively modulating oxidative stress, glucose, and lipid profiles as well as inflammation [20,21]. Kaempferol and narirutin are flavonoids widely distributed in different genera such as *Citrus*, *Brassica*, *Allium*, and *Malus*, among others [21]. Substantial scientific evidence shows that kaempferol blocks cellular neuroinflammation in in vitro and in vivo models [20,22]. Likewise, *citrus* flavonoids have evidenced anti-inflammatory, antioxidant, anti-obesity, and anti-diabetic properties [23]. Narirutin and kaempferol rutinoside, found in grapefruit extract, have been reported to be a negative regulator of inflammation in an obese animal model [23]. Naringenin (aglycone form of narirutin) has been proved to inhibit astroglial activation and recovers the suppression of neurogenesis in the hippocampus of pharmacologic induced-hyperglycemic mice [24]. Also, narirutin is effective in preventing neuroinflammation in a mouse model of Parkinson’s disease [25]. Of note, biological effects of flavonoids are dependent on their bioavailability, regarding their chemical structure, physiological digestion, and microbiota, among others [26]. On the other hand, food components are able to reshape the genome in the uterus by modulating the offspring phenotype later in life [27]. For instance, epidemiological and basic studies have demonstrated that maternal nutrition and metabolic profile play an important role in the programming of the neural circuit that regulates behaviour, which results in long-term effects in the offspring [28]. Together, the role of kaempferol and narirutin as modulators of depression-like behaviour in offspring following maternal hypercaloric programming is unknown.

Here, we seek to determine if obesogenic diets during pregnancy and lactation program depression-like behaviour in female offspring and if flavonoids supplementation during programing might revert behaviour alteration and modulate the inflammatory profile.

## 2. Materials and Methods

### 2.1. Antibodies and Reagents

Narirutin (Cat. SMB00321), kaempferol-3-*O*-glucoside (Cat. 04500585), and imipramine hydrochloride ≥99% (Cat. I7379) were purchased from Sigma-Aldrich, St. Louis, MO, USA.

Primary antibodies were TBK1 (ab40676) (Abcam, Toronto, ON, Canada) and β-actin (2128) (Cell signaling Technology, Danvers, MA, USA). Secondary antibodies were anti-HRP-Mouse IgG (7076S) (Cell signaling Technology, Danvers, MA, USA) and anti-HRP-Rabbit (Inc. sc-2370) (Santa Cruz Biotechnology, Santa Cruz, CA, USA).

### 2.2. Animals and Housing

All the experiments were performed using wild-type female Wistar rats two months old (initial body weight 200–250 g). Animals were handled according to the NIH guide for the care and use of laboratory animals (NIH Publications No. 80-23, revised in 1996). We followed the Basel Declaration to implement the ethical principles of Replacement, Reduction and Refinement of experimental animal models, as it was possible. Our study was approved by the local Animal Care Committee (BI0002). Rats were housed individually in Plexiglas style cages, maintained at 20–23 °C in a temperature-controlled room with a 12-h light/dark cycle. Water was available ad libitum in the home cage. Food availability is described below.

### 2.3. Diets

The standard chow diet formula contained 57% carbohydrates, 13% lipids, 30% proteins, and caloric density = 3.35 kcal/g (LabDiet, St. Louis, MO, USA) (Cat. D12450B). The high-fat diet (HFD) formula was made of 45% calories from fat, including 25.5% carbohydrates, 58% lipids, 16.4% proteins, and caloric density = 4.73 kcal/g (Research Diets, New Brunswick, NJ, USA) (Cat D12451). Cafeteria diet contained a mix of liquid chocolate, biscuits, bacon, fried potatoes, standard diet, and pork pate, based on a 1:1:1:1:1:1:2 ratio, respectively; divided in 39% carbohydrates, 49% lipids, 12% proteins, and caloric density = 3.72 kcal/g. The high sugar diet (HSD) was made of a standard diet and condensed milk based on a 1:1.5 ratio, respectively; divided in 69% carbohydrates, 18.5% lipids, 12.5% proteins, and caloric density = 3.39 kcal/g. We also used a cafeteria diet supplemented with flavonoids, kaempferol-3-*O*-glucoside (15 mg/kg bw) and narirutin (30 mg/kg bw). Of note, it is important to point out that these diets simulate human population feeding habits.

### 2.4. Nutritional Programming by HSD, HFD, Cafeteria, and Cafeteria Diet Supplemented with Kaempferol-3-O-Glucoside and Narirutin

**Figure 1:**
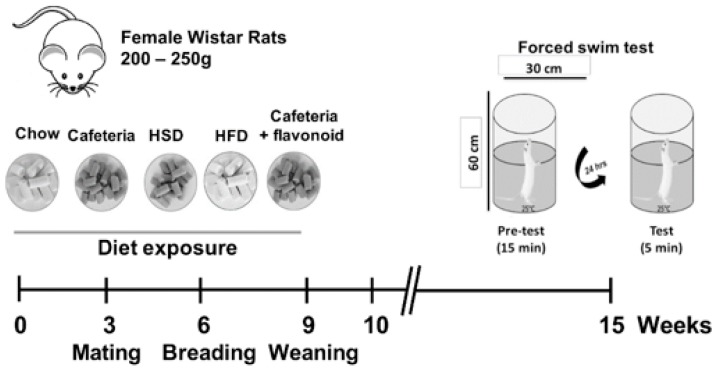
Nutritional programming schedule. Females were exposed to Chow, cafeteria, HSD, HFD, or cafeteria + flavonoids for 9 weeks, as described in Material and Methods. Depression-like behaviour was evaluated by the forced swim test.

Animals were acclimated to the animal facility 7 days prior to the nutritional programming protocol. A total of 32 ten-week-old female Wistar rats were housed in standard conditions as described with ad libitum access to food and water. Female mothers were randomized into five different dietary groups as follows: Standard chow diet (Control, *n* = 4), HSD diet (*n* = 4), HFD (*n* = 4), cafeteria diet (*n* = 6), and cafeteria diet supplemented with flavonoids (*n* = 2). Female rats were exposed ad libitum to specific formula diets three weeks before mating. Rats were mated with age-matched Wistar males for two days and males were removed from the home cage (Figure 1). Pregnant rats were kept on the same diet until offspring birth and lactation. Pregnancy diagnosis was performed in females after mating by vaginal plug. Female rats lacking copulation plugs were returned to the home cage for a second mating. The number of pregnant females, number of male and female pups, and body weight of offspring is shown in Table 1. After 21 days of lactation, female offspring (F1) from the Control (*n* = 14), HSD (*n* = 16), HFD (*n* = 12), Cafeteria (*n* = 16), and Cafeteria + flavonoids (*n* = 16) were exposed to the standard chow diet for 5 weeks before the forced swim test (See Figure 1 for programming schedule details).

### 2.5. Forced Swim Test

We performed the forced swim test as follows. A week prior to the forced swim test, female offspring 8 weeks old were acclimated to the behavioral test room, in order to avoid stress. We used a plexiglass cylinder (60 cm in height and 30 cm in diameter) filled with water (25 ± 2 °C) up to 40 cm. This procedure allows us to keep the subjects away from the bottom of the cylinder, allowing them to move freely.

In order to test the effect of depression-like behavior, rats were intraperitoneally administered with imipramine or vehicle. Chow (*n* = 7), HSD (*n* = 8), HFD (*n* = 5), Cafeteria (*n* = 8), and Cafeteria + flavonoids (*n* = 8) groups of subjects were intraperitoneally administered with (30 mg/kg) 40 min prior to the test session. Also, chow (*n* = 7), HSD (*n* = 8), HFD (*n* = 7), cafeteria (*n* = 8), or cafeteria + flavonoids diet (*n* = 8) groups of subjects were administered with vehicle. Both groups were subjected to a forced swim test in two consecutive days as follows:

Day 1: Training session, allowing the animal to stay in the cylinder for 15 min, forcing him to establish an inescapable aversive scenario. Animals were removed from the cylinder and kept in a plastic worm box for 30 min prior to their transfer to home cages.

Day 2: Test session, the animal remained in the cylinder for 5 min and free movements were identified as follows. Animal’s behaviour during the test session was recorded using a digital camera. At the end of the test, the animal was taken out from the cylinder and dried carefully with a towel. Between each session, the water was drained from the cylinder, the cylinder was dried with a paper towel, and filled again with water (25 ± 2 °C).

We characterized the animal´s behaviour by visualizing each video and identifying three selective behaviours as follows: (1) Swimming, where the animal moved inside the cylinder with the body lying horizontally, without breaking the water surface with the forepaws. Also, swimming was considered when the animal kept the body under the water surface. (2) Climbing, when the animal showed vigorous movements with the forepaws looking like “kicks” above the water surface or against the cylinder wall. (3) Immobility, where the animal floated showing complete immobility or smooth movements, only enough to keep the nose/head above the water surface. We counted each behavior every 5 s during a total test session time (5 min). Results show swimming, climbing, and immobility total counts.

Afterwards, female rats were sacrificed immediately by pentobarbital overdose and guillotine and we isolated brain regions.

### 2.6. Prefrontal Cortex (PFC) and Hippocampus Isolation

The PFC and hippocampus were isolated using micro punches. Tissue was homogenized with lysis buffer (25 mM Tris-HCl, pH 7.5, 150 mM NaCl, phosphatase inhibitors cocktail (Complete, Roche, Mannheim, Germany), protease inhibitors, 1% Triton, and 0.05% SDS), using an ultrasonic homogenizer. Protein quantification was performed by Bradford reagent as described previously [19].

### 2.7. Western Blot

PFC and hippocampus samples were mixed with Laemmli buffer and then heated at 90 °C for 5 min (samples from IP were not heated at 90 °C but were warmed at 37 °C for 30 min) and subjected to SDS–PAGE. Proteins were electrophoretically transferred to nitrocellulose membranes. The membrane was then blocked for 2 h at RT in TBS-T buffer (10 mM Tris, 0.9% NaCl, 0.1% Tween 20, and pH 7.5) containing 5% bovine serum albumin (BSA). Membranes were incubated overnight with primary antibodies at 4°C, anti β-actin antibody (1:1000) and TBK1 (1:2000). The membranes were washed (4 times/5 min) in TBS-T and incubated for 1 h with HRP-conjugated secondary antibody. Proteins were detected by ECL and exposed to X-ray hyperfilms, which were scanned and quantified densitometrically with the 1.31V ImageJ software (Wayne Rasband, National Institutes of Health, Bethesda, MD, USA).

### 2.8. Statistical Analysis

Statistical analyses were conducted using SPSS 13.0 (SPSS Inc., Chicago, IL, USA). Significant changes for offspring survival at 21 days between diet exposure groups was analyzed by statistical significance and determined by log-rank (Mantel-Cox). Statistical differences between the forced swim tests were determined by a Kruskal–Wallis test followed by a Dunn’s Multiple Comparison Test. Western blot data were analyzed using the unpaired Student’s *t*-test. Data are presented as mean ± SD. The significance levels displayed on figures are as follows: * *p* < 0.05, ** *p* < 0.01.

## 3. Results

### 3.1. Nutritional Programming by Diet Exposure Modulates Conception and Survival of Male Offspring

**Figure 2:**
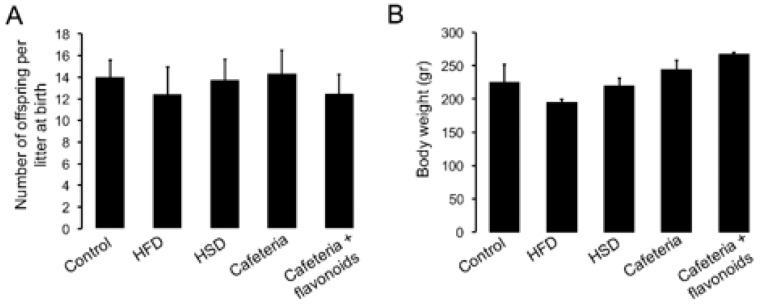
Effect of nutritional programming on birth and body weight in offspring. Diet exposure during pregnancy and lactation was performed as described. (**A**) Number of offspring (males and females) per litter at birth between diet exposure groups and standard chow diet. (**B**) Body weight chance was determined every week and expressed in grams. Graphs show mean ± SD.

Diet exposure during pregnancy and lactation showed specific effects on female conception. HFD and cafeteria diet exposure reduced the female pregnancy after mating, when compared with Chow or HSD exposure (Table 1). Initially, we found that the number of offspring (males and females) per litter at birth do not show significant differences between diet exposure groups and chow diet (Figure 2A). However, HFD exposure decreases the male offspring survival at 21 days when compared to females and control litters (Table 1). No changes in female or male offspring survival at 21 days were found during the HSD, cafeteria, or cafeteria + flavonoids exposure when compared with control litters (Table 1). Finally, time-dependent effect of diet exposure on body weight, including HFD, HSD, cafeteria diet, and the cafeteria-flavonoid supplement diet, at 8 weeks old did not show alterations when compared with standard chow diet (Figure 2B).

### 3.2. HSD Exposure during Pregnancy and Lactation Promotes Insensitivity to the Anti-Depressant Imipramine

**Figure 3:**
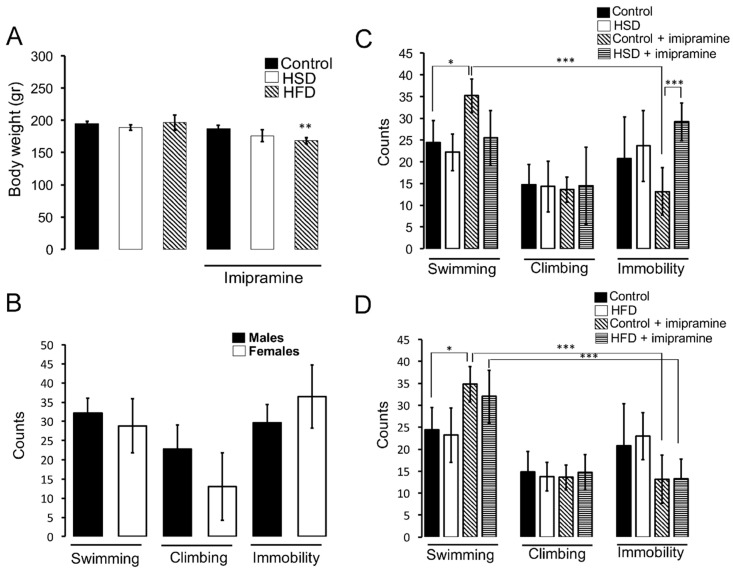
Depressive-like behaviour in HSD and HFD groups. (**A**) Body weight of offspring after i.p. imipramine (30 mg/kg) administration 40 min prior to test session. (**B**) Forced swim test was used to determine swimming, climbing, and immobility events/5 min of male and female rats, as described in Materials and Methods. We video-recorded swimming, climbing, and immobility activities and evaluated each activity as an event which represents a count. Total swimming, climbing or immobility counts/5 min were quantified as shown as mean ± SD. (**C**,**D**) Forced swim test of standard chow diet (*n* = 7), HSD (*n* = 8), and HFD (*n* = 7) subjects. Imipramine (30 mg/Kg) administration to standard chow diet (*n* = 7), HSD (*n* = 8), and HFD (*n* = 5) groups was performed as described. Each count was evaluated during 5 min test. Graphs show mean ± SD. Statistical significance used a Kruskal–Wallis test, followed by a Dunn’s Multiple Comparison Test. * *p* < 0.05, ** *p* < 0.01, *** *p* < 0.001.

We evaluated depressive-like behaviour in males and females using the forced swim test. No changes in body weight were found in HFD and HSD groups following the training session, however, imipramine administration previous to the test correlates with a decrease in total body weight of the HSD group compared to control (Kruskal–Wallis test, followed by a Dunn’s Multiple Comparison Test, ** *p* < 0.01.) (Figure 3A). We also found that, parallel to male, females show correlative swimming, climbing, and immobility counts during the forced swim test (Figure 3B). We identified that HSD, during nutritional programming does not modify swimming, climbing, or immobility counts when compared to standard diet exposure (Figure 3C). Of note, statistical analysis using a Kruskal–Wallis test, followed by a Dunn’s Multiple Comparison Test, showed that intraperitoneal administration of the anti-depressant imipramine in the standard diet group promotes a significant increase in the swimming counts number together with a decrease in immobility behaviour (* *p* < 0.05) (Figure 3C). In addition, HSD exposure during the perinatal period promoted insensitivity to the anti-depressive effects of imipramine, showing no changes in swimming and immobility counts (Kruskal–Wallis test, followed by a Dunn’s Multiple Comparison Test, * *p* < 0.05) (Figure 3C). No changes were detected in climbing during standard and HSD exposure.

Next, we exposed mothers to HFD during pregnancy and lactation and analyzed depressive-like behaviour in the offspring. We found no changes in swimming, climbing, and immobility counts promoted by nutritional fat diet (Figure 3D). Also, in contrast to standard diet exposure, which showed an increase in swimming and a decrease in immobility counts after intraperitoneal administration of imipramine (Kruskal–Wallis test, followed by a Dunn’s Multiple Comparison Test, * *p* < 0.05), we did not find alterations leading to HFD exposure in female offspring (Figure 3D). However, offspring from the HFD group still showed sensitivity to the anti-depressive effect of imipramine, evidenced by a decrease in the immobility count with respect to the swimming behaviour (Kruskal–Wallis test, followed by a Dunn’s Multiple Comparison Test, * *p* < 0.05) (Figure 3D).

### 3.3. Flavonoids Revert the Insensitivity to the Anti-Depressant Imipramine Induced by Nutritional Programming by Cafeteria Diet

**Figure 4:**
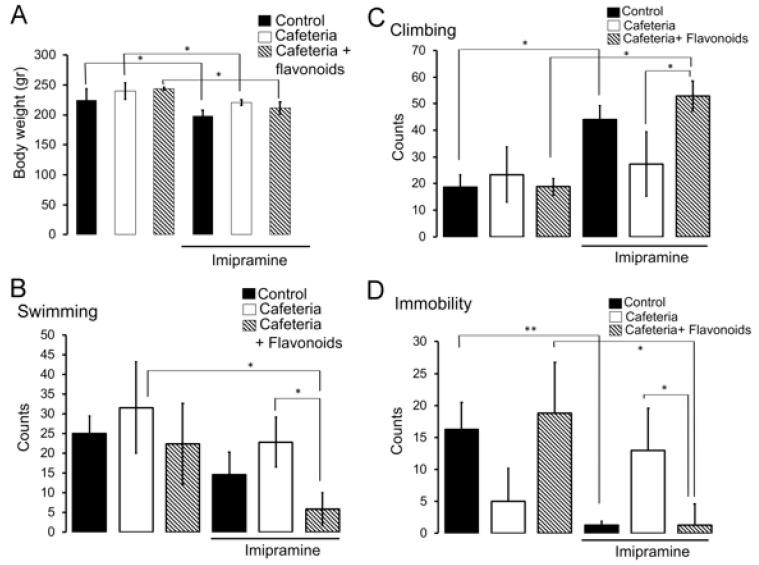
Flavonoids revert depressive-like behavior induced by cafeteria diet exposure. (**A**) Offspring body weight after intraperitoneal imipramine (30 mg/kg) administration 40 min previous to test session. Nutritional programming induced by cafeteria (*n* = 8), cafeteria + flavonoids (*n* = 8), or standard chow diet (*n* = 7) was performed and i.p. imipramine administration to the cafeteria (*n* = 8), cafeteria + flavonoids (*n* = 8), or standard chow diet (*n* = 7), followed by the forced swim test, was accomplished as described previously. Total swimming (**B**), climbing (**C**) or immobility (**D**) counts/5 min were quantified as shown in graphs as mean ± SD. Statistical significance used Kruskal–Wallis test, followed by a Dunn’s Multiple Comparison Test, * *p* < 0.05, ** *p* < 0.01.

The effect of nutritional programming by the cafeteria diet in female offspring on depressive-like behaviour was evaluated. Body weight analysis after imipramine administration to the cafeteria and cafeteria + flavonoids groups was evaluated. We found a significant effect by applying statistic Kruskal–Wallis test, followed by a Dunn’s Multiple Comparison Test of imipramine administration on body weight decrease in cafeteria and cafeteria + flavonoids groups (* *p* < 0.05) (Figure 4A). We also identified that offspring programmed by the cafeteria diet showed no changes in swimming, climbing, and immobility counts when compared with the standard chow diet (Control) (Kruskal–Wallis test, followed by a Dunn’s Multiple Comparison Test, * *p* < 0.05) (Figure 4B–D). The effect of imipramine as a pharmacologic anti-depressive reagent was evidenced in the control standard chow diet group by showing substantial decrease in immobility counts together with an increase in climbing activity and a decrease in swimming behaviour (Kruskal–Wallis test, followed by a Dunn’s Multiple Comparison Test, * *p* < 0.05, ** *p* < 0.01, * *p* < 0.05, respectively) (Figure 4B–D). Of note, we found that cafeteria diet exposure programs female offspring to be unresponsive to the anti-depressive effect of imipramine, showing no changes in swimming, climbing, and immobility counts when compared to the cafeteria group (Figure 4B–D). Notably, the kaempferol-3-*O*-glucoside and narirutin flavonoid cocktail enrichment in the cafeteria diet improves the effect induced by imipramine by showing decrease immobility and swimming counts and increasing climbing activity during the forced swim test (Kruskal–Wallis test, followed by a Dunn’s Multiple Comparison Test. * *p* < 0.05, ** *p* < 0.01, respectively) (Figure 4B–D).

### 3.4. Nutritional Programming by HSD Promotes an Increase in Hippocampal TBK1 Expression of Offspring

**Figure 5:**
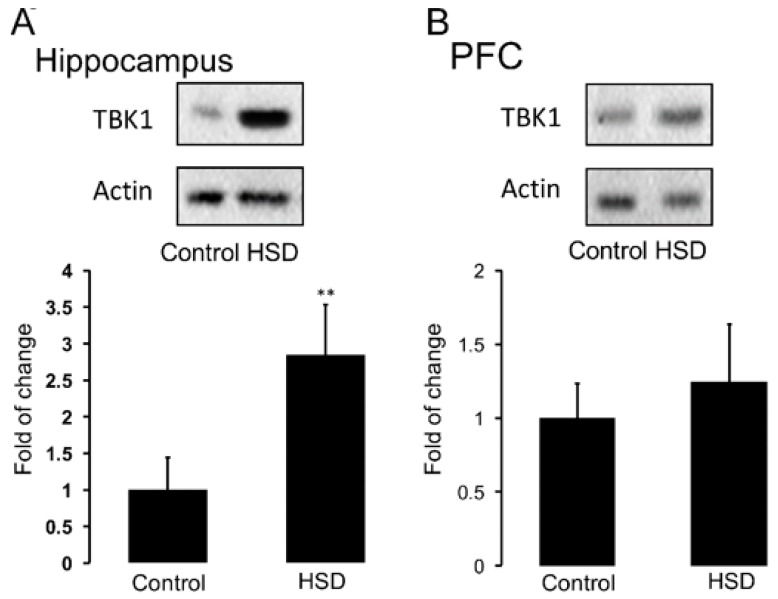
HSD diet exposure during pregnancy and lactation increases TBK1 protein levels in hippocampus. HSD exposure during embryonic development was performed as described. Total protein of TBK1 inflammatory marker in hippocampus (**A**) and PFC (**B**) was determined by Western blot analysis as described in Materials and Methods. Graphs show mean ± SD; *n* = 5–8. Unpaired Student’s *t*-test comparing between groups, ** *p* < 0.01.

Nutritional programming by hypercaloric diet exposure increases inflammatory markers in the brain [13,14,29,30]. We sought to determine if HSD exposure during pregnancy and lactation leads to TBK1 activation (a positive marker of the NF-κB pathway during inflammation). Analysis by the unpaired Student’s *t*-test statistics show that maternal HSD exposure during pregnancy and lactation increases the protein content of TBK1 in the hippocampus of female offspring, when compared to control (** *p* < 0.01) (Figure 5A). No changes in TBK1 protein levels were found in PFC (Figure 5B).

**Figure 6:**
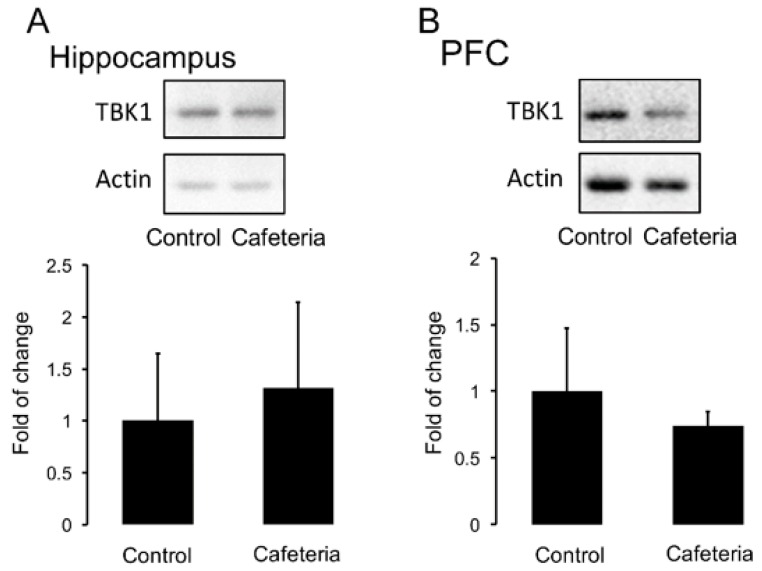
Effect of cafeteria diet exposure during pregnancy and lactation on TBK1 protein levels in the hippocampus. Cafeteria diet exposure during embryonic development was performed as described. Total protein of TBK1 inflammatory marker in hippocampus (**A**) and PFC (**B**) was determined by Western blot analysis as described in Materials and Methods. Graphs show mean ± SD; *n* = 5–8.

We also tested if nutritional programming by maternal cafeteria diet promotes TBK1 expression in the brain of female offspring. Unexpectedly, the cafeteria diet does not alter the protein levels of TBK1 in the hippocampus and PFC samples (Figure 6A,B).

## 4. Discussion

Maternal overnutrition by hypercaloric diet exposure during perinatal periods increase the susceptibility to behaviour alterations in humans and animal models [1,2,3], including long term alteration in anxiety, risk of autism, sensitivity to positive rewards, and depression-like behaviour [4,5]. Here, we identify the effects of nutritional programming by maternal HFD, HSD, and cafeteria diet exposure on depression like-behaviour and its modulation by flavonoids supplementation in female offspring. We show that the cafeteria diet, during the perinatal period, decreases immobility counts and promotes unsensitivity to the anti-depressive effects of imipramine when compared with standard chow diet. Of note, flavonoid supplementation during programming increases mobility and climbing counts similar to control values. Also, we found that nutritional HSD exposure leads to unsensitivity to the anti-depressive effects of imipramine relating to swimming and immobility counts, which correlated with increases in TBK1 inflammatory protein marker in the hippocampus of offspring. These results suggest that in contrast to nutritional programming by HFD exposure, HSD seems to disrupt the sensitivity to pharmacologic inhibition of depression and also that flavonoids recover the anti-depressive effect of imipramine in offspring linked to cafeteria nutritional programming.

External stimuli coordinate fetal programming by regulating neuronal physiology leading to behavioural phenotypes. Nutrition has been recently proposed as a key trigger of programming to coordinate offspring behaviour including anxiety, risk of autism, and sensitivity to positive rewards [1,2,3,4,5,30,31]. Here, we confirm that nutritional programming modulates behaviour and we add new experimental evidence by showing that nutritional programming by HSD exposure fails to show an increase in swimming and a decrease in immobility counts in female offspring, an effect promoted by the anti-depressive imipramine in standard diet animals. The effect of a sugar diet on neuronal function is documented by Lemos, et al. (2016) showing that 9 weeks of exposure to a sucrose-rich diet decreases memory performance and forced swimming test behaviour [32]. Also, male Wistar rats fed with a high-fructose diet during the periadolescent period show increased anxiety-like behaviour and depressive-like behaviour in the forced swim test in adulthood [33]. Of importance, diet effect on alteration of neuronal physiology seems to occur earlier than the effect on peripheral organs. For instance, the effect of HSD exposure on memory performance is not associated with altered hippocampal metabolism but is probably related to modified synaptic plasticity [32].

In contrast to the effect of HSD on depressive-like behaviour, we found that the cafeteria diet, during the perinatal period, decreases immobility counts leading to an increase in swimming and no changes in climbing. Our data is in accordance with Clouard, C, et al. showing failure in behavioural performance induced by a HFD, HSD in piglets [34], and also correlates with increased anxiety-like behaviour and impaired fear extinction retention by HSD exposure to adolescent rats [35]. Also, the cafeteria diet decreases the climbing behavior in the force swim test, indicative of depression-like behaviour [36]. While the molecular mechanism regarding hypercaloric diet programming is unknown, it has been reported that the effects of HSD programming on behavioural alteration are presumably due to a decrease in parvalbumin neurons in the prefrontal cortex [35]. These inconsistent results may be strongly related to strain differences [37], modification on timing, type of malnutrition, the experimental procedures used, and fluctuations in behavior performance.

One of the major achievements from our study was the effect of flavonoid supplementation (kaempferol-3-*O*-glucoside and narirutin) as an important anti-depressive modulator during embryonic development. Flavonoids are positive effectors in biological systems, by their anti-inflammatory properties [21], and are found in food in the aglycone form or mostly as glycosides linked to sugars. The structure of the flavonoid determines its biological properties and is an important factor in the absorption process, which starts in gastric digestion, continuing in the small intestine where the aglycones pass through passive diffusion. Instead, the glycosidic flavonoids are hydrolyzed and finally reach the colon, metabolized by the colonic microbiota [38]. Finally, flavonoids pass to the systemic circulation and tissues and then are excreted in the urine. In this context, depending on their systemic availability and lipophilicity, flavonoids could cross the blood-brain barrier (BBB) and accumulate in specific cells [39]. The glycosidic flavonoids, kaempferol and narirutin, used in our study are widely distributed in different genera such as *Citrus*, *Brassica*, *Allium*, and *Malus*, among others [20]. Plenty of scientific evidence supports the role of kaempferol and naringenin in blocking cell processes, including neuroinflammation in in vitro and in vivo models [20,40].

Maternal diet directly modulates offspring behaviour by affecting the intrauterine environment, however, specific molecular mechanisms are still unknown. Scientific evidence shows that pro-inflammatory profile negatively modulates development in the offspring [41]. In this context, the effects of dietary supplementation with one or more bioactive compounds in dams and their impact on the offspring behaviour have been investigated. Thus, in the present study, supplementation with glycosylated flavonoids during perinatal period improves the depressive-like behaviour phenotype and corrects imipramine sensitivity by increasing mobility and climbing during the forced swim test in offspring exposed to the cafeteria diet. Of note, our data agrees with recent reports showing the antidepressant effect of kaempferol and quercitrin using the tail suspension test, forced swimming test and rota-rod test in a stress group of mice [42].

Hypercaloric diet intake during the perinatal period disrupts behavioural homeostasis in part by its effect on promoting inflammation [10]. Likewise, Winther et al. (2018) show that maternal HFD exposure during perinatal period, might influence offspring neuroinflammatory and stress axis pathways in the hippocampus [43]. Also, Yoshida et al. (2013), evaluate the effect of naringenin supplementation (1%) in rats fed a high-fat diet (60% from fat) and observed a decrease in inflammatory markers [44]. Inflammation might lead to a higher risk of autism, anxiety, and incentive motivation to rewards [14,29,30,31] and it has been identified in depression-like behaviour animal models [10,11,13]. Also, TNF-α release as an inflammatory marker has been documented in patients with idiopathic major depression additional to circulating interleukin (IL)-1, IL-6, and C-reactive protein (CRP) [10,45], which correlates with depression severity [46,47]. In this context, nutrients might be positive modulators of cytokines release from microglia, a brain innate immune cell type. We potentially confirm this hypothesis by showing that chronic HSD exposure during pregnancy and lactation correlates with an increase of the inflammatory TBK1 protein marker in the hippocampus of female offspring. Scientific evidence shows that TBK1 is activated by lipopolysaccharides, double-stranded (ds) RNA, and also by saturated fatty acids [19,48]. Also, recent advances of TBK1 biology in neuronal function have proposed its association to amyotrophic lateral sclerosis (ALS) and fronto-temporal dementia in humans [49,50] by impaired autophagy/mitophagy, leading to retinal cell death in ALS [51]. While the link inflammation-TBK1 activation has not been identified in major depressive disorders, a consistent hypothesis is that an inflammatory profile modulates neuronal function to behavioural arousal. For instance, chronic exposure to elevated inflammatory cytokines might lead to depression [10], whereas acute administration of cytokines or activation of the innate immune system induces anhedonia, anorexia, sleep changes, and failure to social interaction [52,53]. In this context, several studies show the mitigating microglia-mediated neuroinflammation by biocompounds [54]. Of note, kaempferol supplementation in a chronic social defeat stress mice model ameliorates depressive-like behaviour by enhanced anti-inflammatory effects via up-regulation AKT/β-catenin cascade activity [55].

Overall, these data support the hypothesis that metabolic programming by hypercaloric nutrients contributes to behaviour alterations related to depression susceptibility, potentially modulated by flavonoids supplementation.

## 5. Conclusions

Our results support the effect of kaempferol-3-*O*-glucoside and narirutin intake as a potential modulator of susceptibility to depressive-like behaviour in female offspring during the perinatal period. We found that, in contrast to the cafeteria diet, perinatal HSD exposure is potentially effective for increasing TBK1 protein levels in the hippocampus. However, cafeteria diet intake leads to substantial depressive-like behaviour, which is fully prevented by flavonoids supplementation. Our data supports the potential protective role of flavonoids on defective behaviour programmed by maternal obesity during the perinatal period.

## Figures and Tables

**Table 1:** Effect of nutritional programming on breeding and female offspring survival at 21 days.

Diets	Total Female Before Mating	Pregnant Females	Mean Litter Size After Birth	Males at 21 Days of Age	Females at 21 Days of Age	Ratio Male/Female	Total of f1 Females (Behaviour Test)
control	4	4	13.75	28	27	1.04	20
hfd	4	3	14	5	12	0.42 *	12
cafeteria	6	3	13.67	12	20	0.60	20
hsd	4	4	13.75	29	26	1.12	26
cafeteria + flavonoids	2	2	12.5	9	16	0.56	16

Diet exposure during pregnancy and lactation (Control, HFD, Cafeteria, HSD, and Cafeteria + flavonoids) was performed as described. The offspring number per litter was counted and survival was followed until 21 days of birth. The final column shows the number of pups, male and female survival numbers at 21 days, and the total of F1 Females for forced swim test analysis. Statistical significance was determined by log-rank (Mantel-Cox), * *p* < 0.0001.

## Abbreviations

HFD	High fat diet
HSD	High sugar diet
TBK1	TANK binding kinase 1
IL	Interleukin
ANOVA	Analysis of variance
